# Differential Expression of Apoptosis Related Genes in Selected Strains of *Aedes aegypti* with Different Susceptibilities to Dengue Virus

**DOI:** 10.1371/journal.pone.0061187

**Published:** 2013-04-10

**Authors:** Clara B. Ocampo, Paola A. Caicedo, Gloria Jaramillo, Raul Ursic Bedoya, Olga Baron, Idalba M. Serrato, Dawn M. Cooper, Carl Lowenberger

**Affiliations:** 1 Centro Internacional de Entrenamiento e Investigaciones Médicas (CIDEIM), Cali, Colombia; 2 Department of Biological Sciences, Simon Fraser University, Burnaby, British Columbia, Canada; 3 Tekmira Pharmaceuticals Corporation, Burnaby, British Columbia, Canada; 4 Université Nice Sophia Antipolis, Nice, France; 5 Department of Cellular and Physiological Sciences, University of British Columbia, Vancouver, British Columbia, Canada; University of British Columbia, Canada

## Abstract

*Aedes aegypti* is the principal vector of Dengue viruses worldwide. We identified field collected insects with differential susceptibility to Dengue-2 virus (DENv-2) and used isofemale selection to establish susceptible and refractory strains based on midgut infection barriers. Previous experiments had identified higher expression of apoptosis-related genes in the refractory strain. To identify potential molecular mechanisms associated with DENv susceptibility, we evaluated the differential expression of Caspase-16, Aedronc, Aedredd, Inhibitor of apoptosis (AeIAP1) and one member of the RNAi pathway, Argonaute-2 in the midguts and fat body tissues of the selected strains at specific times post blood feeding or infection with DENv-2. In the refractory strain there was significantly increased expression of caspases in midgut and fatbody tissues in the presence of DENv-2, compared to exposure to blood alone, and significantly higher caspase expression in the refractory strain compared with the susceptible strain at timepoints when DENv was establishing in these tissues. We used RNAi to knockdown gene expression; knockdown of AeIAP1 was lethal to the insects. In the refractory strain, knockdown of the pro-apoptotic gene Aedronc increased the susceptibility of refractory insects to DENv-2 from 53% to 78% suggesting a contributing role of this gene in the innate immune response of the refractory strain.

## Introduction

Dengue viruses (DENv), transmitted to humans by infected mosquitoes, cause an estimated 50–100 million cases of Dengue fever (DF), ∼500,000 cases of Dengue Hemorrhagic Fever, and >20,000 deaths per year [1], [2]. DENv transmission has expanded to multiple tropical and subtropical countries and may reach temperate zones due to climate change [3]. There is no available vaccine or effective treatment for DENv. Given the limited success achieved through classical vector control [4], many new strategies to reduce transmission have been proposed including the use of genetically modified vectors [5], [6], [7] or the use of natural symbionts such as Wolbachia [8], [9], [10]. The development of such strategies requires extensive knowledge of the molecular interactions between virus and vector and how these determine vector competence (VC), the intrinsic ability of an arthropod to transmit a pathogen.

A major question is how DENv avoids the innate immune response of the insect vector. Insects recognize unique pathogen-associated molecular patterns (PAMPs) [11], using pattern recognition receptors (PRRs) [12], and activate response pathways such as the IMD and Toll pathways [13] which lead to elimination of parasites through phagocytosis, proteolytic cascades, and synthesis of potent antimicrobial peptides (AMPs) [14], [15]. Most studies have looked at classical responses to parasites that move through the hemocoel to the mouthparts for transmission [16], [17]. More recent studies have addressed the development of intracellular parasites such as DENv, and other arboviruses, in mosquito vectors, and potential roles of specific molecules and pathways that regulate or determine these interactions [17], [18], [19], [20], [21], [22] There is growing evidence that these pathways are not distinct. Components of different immune pathways may function synergistically and may interact with components of apoptosis and other metabolic pathways to determine VC [18], [23], [24], [25]. The VC of *Ae. aegypti* has been studied extensively through the selection of strains with different susceptibilities [18], [26], [27], [28], [29], [30] but no specific genes have been identified as determinants of DENv susceptibility and it is unknown if all geographic strains of *Ae. aegypti* use similar mechanisms and genes against invasion by DENv [18], [30]. The VC of *Ae. aegypti* to a specific virus may be determined by the presence of virus in the salivary glands (Susceptible). Refractory mosquitoes may have infection barriers in the salivary glands or in the midgut where the virus may not be able to enter midgut cells (midgut infection barrier: MIB) or to escape from infected midgut cells (midgut escape barrier: MEB) [28]. Interactions between DENv and *Ae. aegypti* also may be affected by specific genotype-by-genotype interactions [31] and by genetic and environmental interactions that combine to determine VC [32].

Previously we observed a high variation in the VC of mosquitoes caught in various regions of Cali, Colombia [33]. We selected field strains and their progeny for differential susceptibility to DENv-2 using isofemale selection [34]. We used suppressive subtractive hybridization to compare differential gene expression in the midguts of susceptible and refractory strains 48 h after ingesting a bloodmeal containing DENv-2 and compared these data with the responses of a DENv-susceptible laboratory colony [17]. We identified differential expression of genes normally associated with apoptosis [17].

**Figure 1:**
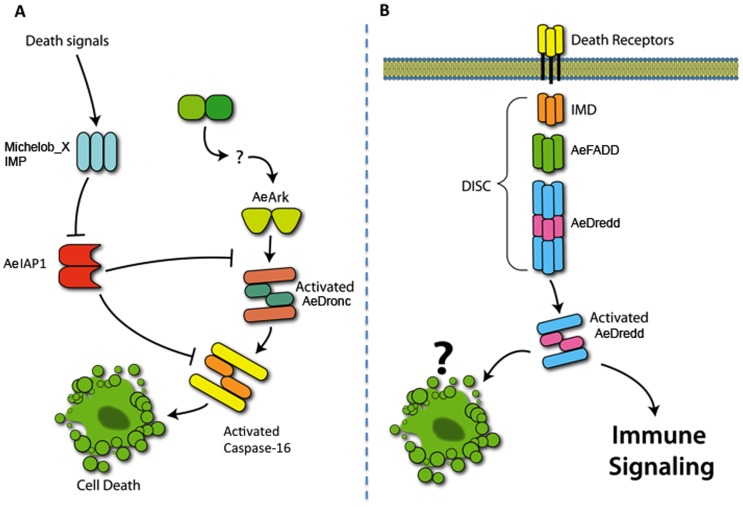
Schematic of cell death and immune signaling pathways in *Aedes aegypti*. **Panel A.** In insects, the primary apoptotic caspase is AeDronc, a caspase-9 homologue with an N-terminal CARD domain for interactions with the caspase adaptor molecule AeARK. AeDronc activation is primarily regulated by the IAP antagonist proteins, Michelob_X and IMP. Together, the IAP antagonists promote cell death by binding to the Inhibitor of Apoptosis Protein, AeIAP1. Once activated, AeDronc will cleave and activate the effector caspase caspase-16. **Panel B.** AeDredd is a death domain containing caspase that contains two death-inducing domains that interact with the caspase adaptor protein, AeFADD (*Aedes* Fas Associated Death Domain containing protein). Both *Drosophila* Dredd and AeDredd were isolated initially as potential inducers of apoptosis. Although apoptotic roles for Dredd have not been ruled out, data suggests that the primary role of Dredd is immune-related.

Apoptosis, among other things, is a directed response to eliminate intracellular pathogens, providing for the death and removal of both the infected cell and pathogen in both vertebrate and invertebrate hosts. Apoptosis comprises a two phase process: a commitment to cell death induced by initiator caspases, followed by an execution phase mediated by effector caspases [35], [36] and is tightly controlled through apoptotic regulators and inhibitors of apoptosis (IAPs) that regulate and promote cell survival or death [36], [37], [38]. Several papers have described the role of apoptosis as a defence against viruses and other pathogens [39], [40], apoptosis-like activity in infected mosquitoes [41], [42], [43] or the identification of apoptosis-related genes in microarray studies [18], [30]. We characterized some of the molecules involved in the *Ae. aegypti* apoptotic pathway [23], [44], [45] but the role of apoptosis as an anti-Dengue immune response remains unclear. Some of the molecules studied in this manuscript and their putative pathways and interactions are indicated in Fig. 1.

We report here the differential expression of selected genes in field-derived strains of *Ae. aegypti* from Colombia that are susceptible (Cali-S) or refractory through a midgut infection barrier (Cali-MIB) to infection with DENv-2. Upon ingesting DENv-2, Cali-MIB expresses significantly higher levels of pro-apoptotic genes (Aedronc, Aedredd, Caspase-16) than Cali-S whereas both strains express similar levels of apoptosis inhibitors (AeIAP1).

## Materials and Methods

### Ethics Statement

All insects were exposed to Dengue viruses through an artificial membrane feeder. Insect colonies were fed on guinea pigs at SFU under Animal Care protocol 1000B-02 approved by the SFU Animal Care Committee following the guidelines of the Canadian Council of Animal Care, or rabbits at CIDEIM under protocols approved by the CIDEIM institutional review committee for research in animals (CICUAL) under Federal Wide Assurance number A5643-01, of the US Department of Health and Human Services.

### Mosquitoes and strain selection

The field collection of larvae, exposure to DENv-2, phenotype determination and strain selection, to establish Cali-S and Cali-MIB were described previously [17], [34]. Briefly, laboratory colonies were established from field collected larvae and pupae from larval habitats in 5 locations at least 10 km apart in the city of Cali, Colombia. The mosquitoes were maintained under standard laboratory conditions: 26±2°C, 70% relative humidity, and a 12∶12 hour light∶dark cycle. Larvae were maintained in plastic containers at a density of 300 larvae/2L of water and were fed daily with 2 mL of a stock solution of beef liver (DIFCO™ Liver - 8 g/400 mL). Adults were fed with a 10% sugar solution. Bloodfeeding was done through an artificial feeder using a pig intestine membrane and defibrinated rabbit's blood. The blood was tested for the presence of dengue virus after every feeding process. Eggs from females showing the S or MIB phenotype were combined. Selection by exposure to DENv-2 was done every second generation [34].

### Virus Strain

Dengue-2 virus New Guinea C strain, freshly grown in *Aedes albopictus* C6/36 HT cells, was used in oral challenges. Infected cells were incubated for 14 days at 32°C in L15 medium supplemented with 2% heat-inactivated fetal bovine serum, 1% penicillin/streptomycin, and 1% L-glutamine [46]. Virus and cells were harvested and collected in a 15 mL conical centrifuge tube. Aliquots of the infected cell suspension and the mixture of blood and virus before and after the infection process were titred as described [27]. Titers in the cell suspensions ranged from 10^8^ to 10^8.5^ TCID_50_/mL in all oral challenges. The viral suspension used to feed mosquitoes also was microinjected intrathoracically into mosquitoes to serve as positive controls for indirect immunofluorescence (IFI) studies.

### Mosquito infection and virus titration

Five to seven day old female Cali-S and Cali-MIB *Ae. aegypti*, that had been starved for 12 h, were exposed for 30 minutes to an infectious bloodmeal consisting of a 1∶1 mixture [47] of defibrinated rabbit blood and the dengue virus suspension via a water-jacketed membrane feeder using a pig intestine. Fully engorged females were separated and housed in groups of 20 in cartons covered in mesh and with access to a 10% sugar solution *ad libitum*. Infections were done in BSL-2+ facilities. The presence and concentration of virus to which the females were exposed was determined in uninfected rabbit blood, virus suspension, and the mixture of blood and virus at the beginning and end of the exposure period as described [27]. IFI was used to determine the percentage of infections in the head and midgut of each female as described [48]. The barriers to virus dissemination were determined 13 d post infection as described [27], [28].

### Tissue dissection and RNA isolation

At 0, 8, 24, 36, 48, 72 and 120 h post ingestion of blood or blood+DENv-2, midguts and carcasses (fatbody) from individual mosquitoes from the Cali-S (F_16_) and Cali-MIB (F_13_) strains were dissected on a chilled table and carefully rinsed in cold DEPC-PBS to remove traces of the blood meal. Each midgut was stored separately in a 1.5 ml tube with 100 µL of RLT Lysis Buffer (Qiagen, Valencia, CA) and its corresponding carcass was stored individually in RNAlater (Ambion, Austin, TX) at −20°C. Total RNA extraction from individual midguts and carcases was performed using RNeasy Mini Kit (Qiagen, Valencia, CA). Total RNA was quantified using a NanoDrop Spectrophotometer ND-1000 (NanoDrop, Wilmington, DE).

### cDNA synthesis and detection of infection with Dengue-2 virus in tissues

RNA (90 ng) was reverse transcribed in a 20 µL reaction mixture containing 1× first strand buffer (50 mM Tris-HCl (pH 8.3), 75 mM KCl, 3 mM MgCl2), 0.005 M DTT, 0.5 mM of dNTPs mix, 0.5 pmol/µL of primer D2 (5′- TTGCACCAACAGTCAATGTCTTCAGGTTC-3′) and 0.625 units of Superscript II Reverse Transcriptase (Life Technologies, Grand Island, NY). Reverse transcription was conducted at 42°C for 60 min and 95°C for 5 min. A nested PCR protocol, modified after Lanciotti et al [49], was used to detect the virus in individual midguts [17] in 50 µl PCR reactions containing 1× PCR buffer (50 mM KCl, 10 mM Tris-HCl (pH 9.0), 1.5 mM MgCl2, 250 µM of each dNTP, 0.5 pmol/µL of primers D1 (5′- TCAATATGCTGAAACGCGCGAGAAACCG-3′) and D2, and 0.05 U/µL of Taq DNA polymerase (Life Technologies, Grand Island, NY). PCR was performed with the following parameters: 95°C for 1 min; 30 cycles of 94°C for 45 s, 58°C for 45 s, and 72°C for 1 min; and a final extension at 72°C for 7 min. A second-round PCR was run with a 1∶100 dilution from the first PCR reaction. PCR was performed under the same conditions used for the primary PCR with the following modifications: primer D2 was replaced with the Dengue-2 virus-specific primer TS2 (5′- CGCCACAAGGGCCATGAACAG-3′) and 35 amplification cycles were used. PCR products were resolved by 2% agarose gel electrophoresis, stained with ethidium bromide and visualized under UV light.

### Pool generation

The detection of DENv in the section above allowed us to determine the phenotype of individual females. Due to the presence of DENv in the blood bolus within the midgut we could only determine the refractory or susceptible phenotype for time points ≥36 h. RNA from females with the same phenotype was pooled by timepoint. Biological samples of 10 midguts or 5 carcases from each strain and time point were generated.

### Real time quantitative PCR assays

Based on our preliminary and published [17] data, and other recent studies that described the potential role of key immune- and apoptosis-related molecules in *Ae. aegypti*-DENv interactions [20], [50] we selected five candidate genes for evaluation in the midguts and carcasses of Cali-S and Cali-MIB strains of *Ae. aegypti*. These included Argonaute-2, an important component of the RNAi process proposed to reduce or modulate arbovirus replication [51], [52], [53]; Aedronc, an initiator caspase [45]; Aedredd, an initiator caspase [44]; Caspase-16, an effector caspase [20]; and AeIAP1, an inhibitor of apoptosis [17]. Accession numbers for these genes are listed at the end of the manuscript.

For cDNA synthesis, 100 ng of total RNA/time point were reverse transcribed in a 20 µL reaction mixture containing 5× first strand buffer (50 mM Tris-HCl (pH 8.3), 0.1 M DTT, 10 m*M* of each dNTP 50 ng of Oligo(dT) primer (5′-CGGGCAGTGAGCGCAACGTTTTTTTTTTTTTT-3′) and 200 units of Superscript II Reverse Transcriptase (Life Technologies, Grand Island, NY). Reverse transcription was conducted at 42°C for 50 min and 70°C for 15 min. The resulting cDNA was used in the subsequent qPCR reactions. The primers used in all qPCR reactions are shown in Table S1. qPCR conditions used were: 95°C: 2 min, 40 cycles of 95°C: 10 s, 60°C: 15 s, 72°C: 20 s in 25 µL reactions using PerfeCTa SYBR Green Super-Mix (Quanta BioSciences, Gaithesburg, MD) in a Rotor-Gene 3000 (Corbett Research, Sydney, Australia). Gene expression profiles of both refractory and susceptible strains were performed for each gene of interest and normalized to a housekeeping gene, ß-actin.

### qPCR Analysis

**Figure 2:**
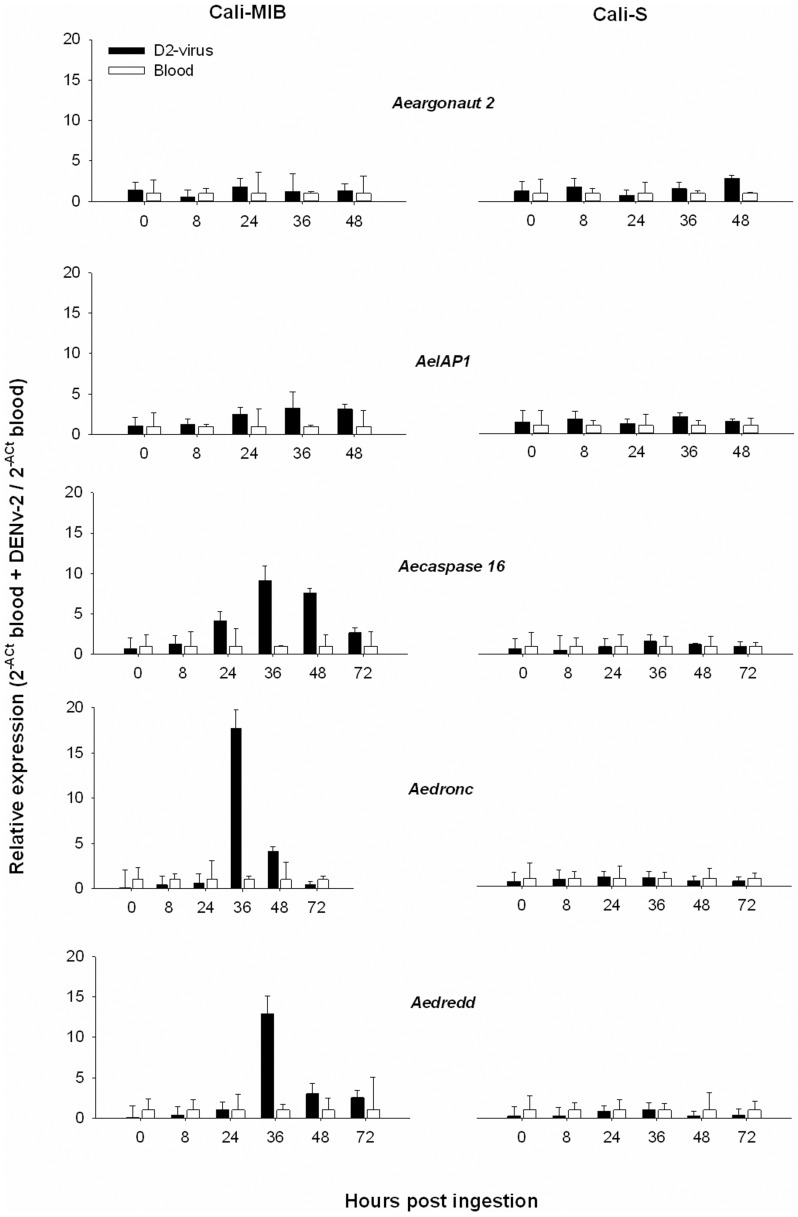
Within Strain Comparisons: Relative expression of Argonaute-2, AeIAP1, caspase-16, Aedronc and Aedredd in the midguts of *Aedes aegypti* in strains that are Refractory (Cali-MIB: Left Panel) or Susceptible (Cali-S: Right Panel) to Dengue virus in the presence (black bars) or absence (white bars) of Dengue virus-2 in the bloodmeal. The expression levels in the bloodfed samples were arbitrarily set at 1 and the expression levels in the presence of the virus represent fold-differences from the susceptible controls. The bars were geometrically adjusted (2^−ΔCT^)+SD.-2^−ΔCT^.

**Figure 3:**
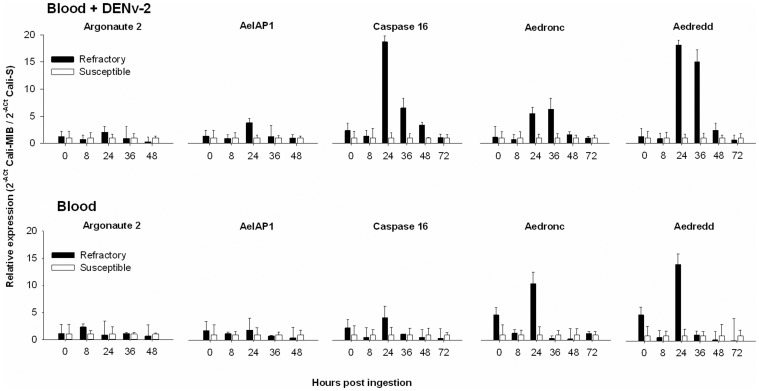
Between Strain Comparisons: Differential expression of Argonaute-2, AeIAP1, Caspase-16, Aedronc, and Aedredd in the midguts of Resistant Cali-MIB (black bars) and Susceptible Cali-S mosquitoes (white bars) at the same timepoints after exposure to a bloodmeal containing DENv-2 (top panel) or blood alone (bottom panel). In each pairwise comparison the expression level in the Cali-S strain was arbitrarily set at 1 and the expression levels in Cali-MIB strain represents fold-differences in expression within that pair. The bars are geometric adjusted (2^−ΔCT^)+SD.

Real-time quantitative PCR results were analyzed using described methodologies [54], [55]. We normalized expression levels using an internal control (ß-actin) to generate ΔCt values. We used the 2^−ΔΔCt^ method using the untreated sample (Time 0) as the second calibrator to measure fold changes. We compared gene expression within each strain exposed to blood+DENv-2 or blood alone using 2^−ΔCt^_D2virus_/2^−ΔCt^_Blood_ (Figure 2); and between strains (Cali-MIB vs Cali-S) after exposure to blood or blood+DENv-2 using 2^−ΔCt^_Cali-MIB_/2^−ΔCt^_Cali-S_ (Figure 3). The results are presented as the means and standard deviations of two-three independently generated cDNAs assayed at least twice and where each sample was run in triplicate.

Statistical analysis was performed using multiple linear regression with the response variable being ΔCt (Ct (sample) - Ct calibrator (ß-actin)) with the independent factors being time, treatment (Blood vs Blood+DENv-2), and strain (Cali-S vs Cali-MIB). Coefficients comparing ΔCt values estimate the corresponding ΔΔCt values. The model was fit with robust standard errors, allowing for clustering within samples. Analysis was performed using Stata 9.0. A two-sided significance level of 0.05 was used.

### Gene knockdown studies using RNAi

We generated DNA templates of 400–500 bp containing a T7 promoter site on each strand for each target gene. One µg of this template was used for *in vitro* transcription at 37°C for 2–6 h to generate dsRNA following manufacturer's instructions (MEGAscript RNAi, Ambion, Austin, TX). The remaining DNA template and ssRNA were degraded by DNAse I and RNAse treatment at 37°C for 1 h. The dsRNA was purified by centrifugation through a solid-phase adsorption system and eluted in 100 uL of 10 mM Tris-HCl pH 7, 1 mM EDTA. We quantified the dsRNA by spectrophotometry and verified its integrity and the reaction efficiency on a 1% agarose gel. Finally, we precipitated dsRNA with ethanol and ammonium acetate and resuspended in 10 mM Tris-HCl pH 7, 1 mM EDTA to a final concentration of 2 µg/mL.

Different amounts of dsRNA (50–200 ng) were injected intrathoracically [23], and tissues collected at various times to measure the kinetics, duration, and knockdown efficiency using qPCR. Subsequently Cali-MIB mosquitoes were injected with 100 ng of the target dsRNA; Caspase-16, Aedronc, or the Nautilus control gene (Flybase: FBgn0002922) a myogenic regulatory gene from Drosophila as a control for the process of injecting dsRNA. These mosquitoes, along with non-injected controls, were exposed to DENv-2 24 h later in groups of 20. A subsample of mosquitoes was evaluated 48 h post-ingestion of virus for gene expression levels. The remaining mosquitoes were maintained in the insectary for the 13-day extrinsic incubation period for DENv-2, when the heads and midguts were scored for phenotype using IFI [33], [56] to determine if we had affected virus establishment and dissemination. We used a Chi^2^ analysis to compare the effects of gene knockdown on the prevalence of infection in knockdown vs non-injected controls.

## Results

We evaluated gene expression in female *Ae. aegypti* in the Cali-S strain (F_16_) in which 96.4% of the female showed the susceptible phenotype (salivary glands positive for virus) and the Cali-MIB strain (F_13_) in which 53% of the female were susceptible and 47% had the refractory phenotype MIB. The titer of the DENv-2 preparation used was monitored throughout all selections and ranged from 10^8^ to 10^8.5^ TCID_50_/mL at the beginning of virus exposure and from 10^7.2^ to 10^7.4^ TCID_50_/mL at the end of the exposure period. All rabbit blood was free of DENv infection.

### Gene Expression

The expression patterns of the selected genes in both strains were compared within a strain in response to bloodmeals containing blood or blood+DENv-2, and then between strains at the same timepoints after receiving the same challenge.

Within strain comparisons are shown in Fig. 2. In the Cali-S strain, there were no major differences in the expression levels of any of the selected genes in response to meals containing blood, or blood+DENv-2. Within the Cali-MIB strain, there were small but not statistically significant differences in expression levels of Argonaute-2 and AeIAP1 (Figure 2, Table 1). Caspase-16 increased expression 4.2-fold at 24 h, 14.5-fold at 36 h and 7.9- fold at 48 h, Aedronc increased 65-fold at 36 h and 3.8-fold at 48 h and Aedredd expression increased 13-fold at 36 h after challenge with DENv-2 (Figure 2). Using the 2^−ΔΔCt^ method we observed overall differences in gene expression over time of each gene when each strain was challenged with blood or blood+DENv-2 (data not shown). The greatest differences in gene expression were observed between 24 and 48 h especially when the strain Cali-MIB was challenged with DENv-2 (Figure 2). Multiple regression analysis demonstrates significant temporal differences in gene expression compared with time 0 in all genes with the exception of Aedredd (Table 1), due to a high variation in Aedredd expression at time 0. Multiple regression analysis did not demonstrate overall significant differences within strains challenged with blood or blood+DENv-2, except in the case of Caspase 16 (Table 1). When the time points are analyzed independently, however, there were significant differences in the expression of caspase genes between 24 and 48 h when the Cali-MIB was exposed to blood+DENv-2 (Fig. 2).

**Table 1:** Gene expression ratios comparing midgut gene expression of Argonaute-2, AeIAP1, Caspase-16, Aedronc, and Aedredd over time, compared with time zero (ΔCt), in Cali-S and Cali-MIB strains exposed to blood or blood+DENv-2, as well as between-strain ratios.

		Gene Expression Ratio				
		(95% confidence interval)				
		Argonaute-2	AeIAP1	Caspase 16	Aedronc	Aedredd
**Hours post ingestion**	**0** a	1	1	1	1	1
	**8**	2.9	0.5	0.03	10.7	1.3
		(2.03–4.2) P<0.01	(0.4–0.7) P<0.01	(0.01–0.08) P<0.01	(3.8–30.5) P<0.01	(0.3–4.4) P = 0.67
	**24**	19.4	1.6	0.04	14.2	1.7
		(13.7–27.5) P<0.01	(1.0–2.6) P = 0.04	(0.01–0.08) P<0.01	(4.5–44.5) P<0.01	(0.4–6.6) P = 0.38
	**36**	6.75	0.68	0.19	5.5	1.1
		(5.2–8.72) P<0.01	(0.4–0.9) P = 0.04	(0.09–0.43) P<0.01	(1.5–19.8) P<0.01	(0.2–4.3) P = 0.88
	**48**	88.2	7.71	0.33	77	2.6
		(46.8–165.9) P<0.01	(4.1–14.4) P<0.01	(0.17–0.64) P<0.01	(23.3–260.2) P<0.01	(0.6–10.3) P = 0.16
**Within strains (B vs B+v)** b		1.03 (0.5–1.8) P = 0.907	1.7 (0.9–3.1) P = 0.063	2.8 (1.2–2.8) P = 0.012	1.25 (0.4–3.8) P = 0.689	1.17 (0.3–3.9) P = 0.792
**Between strains (Cali-S vs Cali-MIB)**		0.82 (0.5–1.2) P = 0.339	1.2 (0.8–1.8) P = 0.253	4.43 (2.2–8.76) P = 0.01	2.54 (1.2–5.3) P = 0.014	4.8 (1.9–12.0) P = 0.01

Within strains, the treatments blood and blood+DENv-2 are also compared. ΔCt values were estimated from the multiple regression analysis and the expression ratios were estimated by exponentiating (2^−ΔCT^).

a Expression ratio 1 as this is the reference category.

b Blood vs Blood+DENv-2.

Between strains comparisons of gene expression (Cali-S vs Cali-MIB) after exposure to blood or blood+DENv-2 in midguts are shown in Figure 3. In the absence of DENv-2 in the midguts, expression levels of Argonaute-2, and AeIAP1 were not significantly different between the 2 strains (Fig. 3). In contrast, Caspase-16 activity was 3.8-fold higher in the Cali-MIB strain at 24 h. Aedronc expression was 4-fold higher at 0 h, and 11-fold higher at 24 h, and Aedredd expression levels were 5-fold and 14-fold higher in the Cali-MIB strain at 0 and 24 h respectively.

In the presence of DENv-2 in the midguts, there were minor increases, 2.1 fold and a 2.3 fold, in expression of Argonaute-2 in the Cali-MIB strain at 24 and 36 h. AIAP1 expression was increased 3.8 and 2.2 fold in the Cali-MIB strain at 24 and 36 h respectively. With the caspases, there were significant increases in expression of caspase-16 at 24 h (18-fold), 36 h (11-fold), and 48 h (3.4-fold). Elevated levels of Aedronc expression were observed at 24 h (5-fold), and 36 h (24-fold) and Aedredd expression was higher at 24 h (18-fold) and 36 h (15-fold) post-exposure to virus (Figure 3). Multiple regression analysis demonstrated significant differences between treatments with Caspase 16, Aedronc and Aedredd expression in Cali-MIB mosquitoes after challenge with DENv-2 (Table 1). In the carcasses, in the absence of DENv-2, we found no significant differences in gene expression between the strains (data not shown). In the presence of DENv-2 there was a small, but statistically insignificant, increase in Argonaute-2 and AeIAP1 expression at 120 h. Multiple regression analysis in carcasses demonstrate significantly higher levels of Caspase-16 (ratio = 7.5, 95% confidence intervals (CI) 1.8–31.2, p = 0.007) and Aedronc (2.2, CI 1.0–5.0, p = 0.045) expression in the Cali-MIB strain after exposure to DENv-2 especially at 120 h (data not shown).

### Gene knockdown studies

We knocked down the expression of specific genes to determine their contribution to the S or MIB phenotype. We knocked down AeIAP1 in the susceptible *Ae. aegypti* Liverpool strain and in Cali-S to interfere with caspase inhibition, thereby converting the phenotype from S to R. While we were successful in knocking down AeIAP1 by >70%, the phenotype was lethal: all mosquitoes died within 72 h (data not shown).

**Figure 4:**
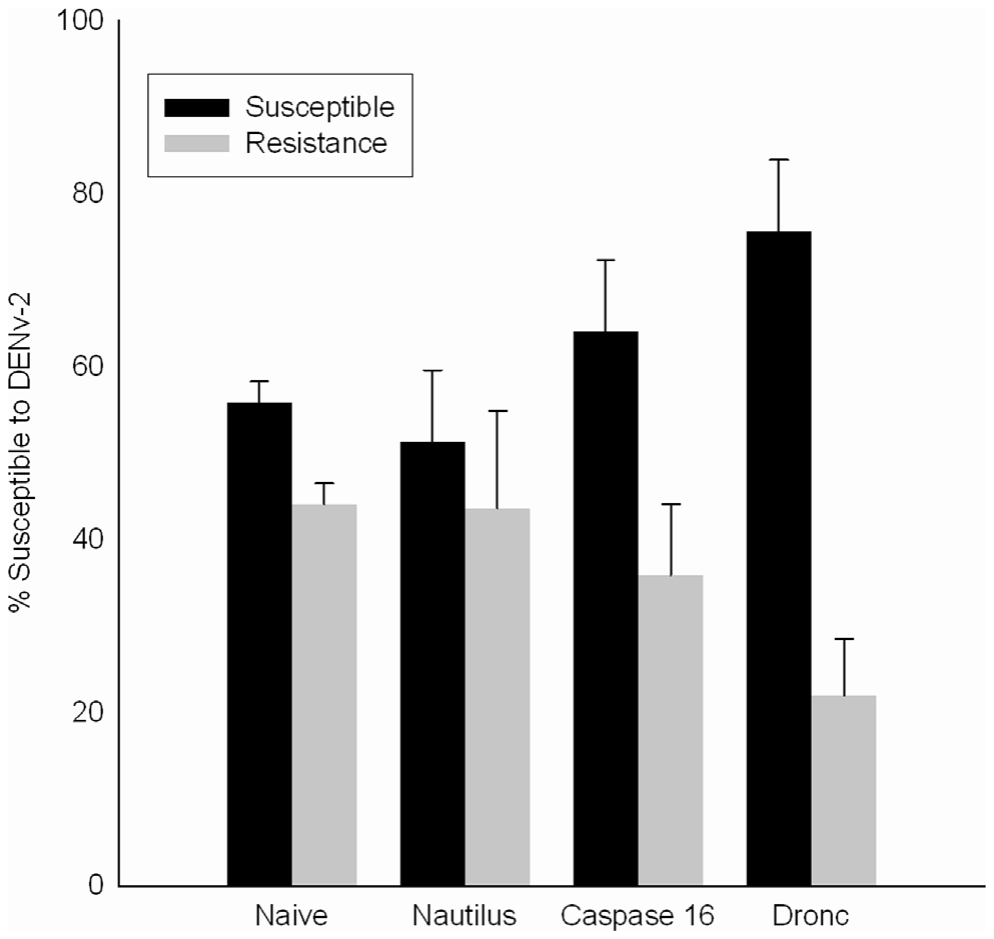
Effects of RNAi knockdown of Aedronc, Caspase-16 or Nautilus on the development of DENv in the refractory Cali-MIB strain of *Ae. aegypti*. The expected proportions in the Cali-MIB colony (53% Susceptible: 47% Resistant) were maintained in naïve and Nautilus injected insects. Knockdown of Caspase-16 and Aedronc using RNAi increased the proportion of susceptible mosquitoes to 62% in Caspase 16 Kd insects (Chi^2^ =  = 0.39; p = 0.53) and 78.6% in the Aedronc Kd insects (Chi^2^ = 3.9; p = 0.03). The numbers in brackets above each pair of bars indicates the # of mosquitoes in 3–5 replicates on which these summaries are based. The * indicates significant difference between Aedronc knockdown and naïve and Nautilus injected controls.

In the Dengue-refractory Cali-MIB (F_21_) strain the microinjection of 100 ng of Caspase-16 or Aedronc dsRNA reduced transcript levels by ∼80% 48 h later. Higher concentrations of dsRNA gave similar results but caused mortality. Cali-MIB mosquitoes that had been injected with 100 ng dsRNA to knockdown Caspase-16, Aedronc, or Nautilus, a *Drosophila melanogaster* MyoD-related gene to control for the injection of dsRNA, and naïve (not microinjected) controls were exposed to a bloodmeal containing DENv-2 24 h after microinjection. These mosquitoes were scored for phenotype after the 13-day extrinsic incubation period for DENv-2 using IFI [30], [31]. In mosquitoes injected with dsRNA to knockdown Caspase-16 or Aedronc, the proportion of mosquitoes that were susceptible to DENv-2 increased from the expected 53% (Naïve) to 62% and 82% respectively (Fig. 4). Chi^2^ analysis on Caspase-16 indicated no significant effect on DENv development (chi^2^ =  = 0.39; p = 0.53) whereas the knockdown of Aedronc significantly altered the susceptibility of the Cali-MIB strain (chi^2^ = 3.9; p = 0.03) indicating that reducing the expression of at least one caspase gene increased pathogen success.

## Discussion

The antibacterial and antifungal responses of insects have been much better analyzed than the antiviral responses; we understand how microbes are recognized as nonself and which pathways are activated [57], [58]. Intracellular viruses may be inaccessible to the same responses used to eliminate larger parasites. More recent studies on immune signalling in response to virus infections in insects have studied which signalling pathways are activated, and identified genes that are differentially expressed in response to virus infections [17], [18], [21], [22], [30], [59], [60], [61]. The response by insects to all viruses, however, may not be equal; the same virus may elicit different responses in different insects and different viruses (alphaviruses vs flaviviruses) may elicit different responses in a single insect species [22], [60]. Arbovirus infections may activate classic antimicrobial immune pathways, including Toll, JAK/STAT, Wnt, and Imd/Jnk [18], [22], [30], [62], [63], but the components or molecules responsible for the activation are still unknown. There is growing evidence that immune pathways function as networks of pathways and interact with each other [18], [30]. Some molecules may function in multiple pathways; the initiator caspase Dredd and its adaptor, Fadd, function in apoptotic and also in the IMD pathway [50], [64], [65]. Increases in Dredd may induce expression of the transcription factor rel which also contributes to the expression of pro-apoptotic pathways. Previously we showed that a targeted knockdown of Aefadd in *Ae. aegypti* reduced the expression of defensin and cecropin transcripts to negligible levels, making them susceptible to microbial infection [23]. Understanding these indirect effects becomes more important in light of recent data indicating that the AMP cecropin can regulate viral replication and ultimately viral load in the salivary glands of DENv infected insects [66]. How molecules that function in multiple pathways are regulated so as not to activate all pathways is unknown, as are the initial mechanisms by which arboviruses, or infected cells, are recognized. It has been suggested that arbovirus infection of mosquito cells triggers apoptosis only when viral loads exceed a specific threshold [67] but our data suggest the apoptotic response may be mosquito or strain specific, and may help determine strain-specific susceptibility to DENv.

We did not determine the mode of inheritance of the susceptible or refractory phenotypes. In the Cali-S strain the proportion of individuals with this phenotype increased progressively with generations. In contrast, there was greater variation in the selection results for Cali-MIB that only reached 44% refractoriness in generation 16 [34]. Our data, and other studies, suggest that VC is a product of multiple genes acting additively or in a dominant manner [26], [29], [68]. These results suggest that *Ae. aegypti* is an excellent model for the study of selection processes that will allow an evaluation of the genetic complexities of specific aspects of VC.

The differences observed in AeIAP1 and Argonaute-2 expression between the Cali-MIB and Cali-S strains were not statistically different, suggesting that these genes do not play a major role in determining the phenotype of our strains. In the presence of DENv-2, however, we did see significant increases in the expression levels of caspases (Caspase-16, Aedronc and Aedredd) in the Cali-MIB strain compared with the Cali-S strain. It should be noted that in the susceptible Cali-S strain the expression of the pro-apoptotic genes did not differ significantly when this strain was fed on blood, or blood+DENv-2 (fold difference <2 in all cases). The data suggest that increased expression of pro-apoptotic genes might contribute to the *Ae. aegypti* innate immune response to DENv-2 infection in Cali-MIB. Whether the increases we measured in AeIAP1 are insufficient to regulate the significant increases in Caspase-16 and Aedronc is unknown. The increase in caspase gene expression occurs at the time when DENv-2 has become established and is replicating in the mosquito midgut cells (24–48 h) or the fatbody (72–120 h) in these strains.

The role of apoptosis as an antiviral response has been proposed in several insect-virus relationships [69], [70], [71] and apoptosis-related genes have been identified in transcriptome studies [18], [21], [30]. We observed a differential expression at timepoints that correspond with the virus replicating within midgut cells 24–48 h post ingestion in our strains. Apoptotic activity has been reported in the midguts of *Culex pipiens pipiens* that confers refractoriness to West Nile Virus (WNv) [43] and in early infections of *Ae. aegypti* larvae exposed to *Culex nigripalpus* nucleopolyhedrovirus [69]. Differential expression of apoptosis-related, and many other genes, was demonstrated using microarrays with laboratory strains (Moyo-S and Moyo-R) of *Ae. aegypti* originally selected for resistance to *Plasmodium* sp. [18]. Differentially expressed genes at 3 and 18 h post ingestion of DENv included genes associated with cell division and apoptosis as well as genes related to several signal transduction pathways [18]. Similarly, specific classes of genes were found to be over or under expressed in *Ae. aegypti* (Rockefeller strain) infected with West Nile, dengue, and yellow fever viruses [30]. Differences in the levels of expression data obtained and even the gene clusters identified may be related to the timepoints at which samples were collected (3 and 18 h post infection in [18], 24, 48 and 168 h in [30], and 12–72 h in this study). Results also may reflect differential development rates (extrinsic incubation period) of different DENv strains in different vector strains [72]. Nevertheless, in all studies, many of the same molecules and pathways have been identified as a result of infection with DENv. How apoptosis is initiated, what molecules are used, how information and responses are shared and regulated within the overall networks of differentially regulated and expressed immune responses in mosquitoes, and how viruses inhibit these processes are not well understood [18]. Studies have expressed pro-apoptotic inducers (Michelob_x, reaper) [69] or the antiapoptotic baculovirus protein p35 [73]. Initial virus production was not significantly altered by these molecules leading to the conclusion that apoptosis was not effective at reducing early virus production. Overexpression of IAP antagonists Michelob-x and IMP can induce apoptosis in mosquito cells while silencing them can attenuate apoptosis [74]. We did not find any differences in the transcription of Michelob_x in Cali-S and Cali-MIB in response to DENv-2 infection at any timepoint evaluated (Ursic, Ocampo and Lowenberger, unpublished data), but we, and others [18] do find immune activation very soon after ingestion of DENv. Determining whether early apoptosis is capable of eliminating DENv establishment and replication will require a measurement of virus titers throughout the infection and TUNEL and *in situ* nick translation studies on DENv-infected midguts of our strains.

RNAi has been reported as a major innate mechanism to modulate or regulate viruses in insects [51], [53], [75], [76] and Argonaute-2 has been implicated in this process. However, we did not find significant differences between Cali-S and Cali-MIB in the expression of Argonaute-2, although both strains showed small increases in Argonaute-2 expression (2–3 fold) when they were exposed to DENv-2. We do not know if this level of expression is sufficient to modulate virus replication as reported elsewhere [53], [77]. Our data suggest that RNAi, as measured by the expression of Argonaute-2, does not differ significantly between our strains, and it does not appear that this mechanism is solely responsible for virus refractoriness in the Cali-MIB strain.

We used RNAi to knockdown expression of pro-apoptotic genes in the Cali-MIB strain to evaluate their functional role in limiting DENv-2 establishment, and proposed that gene knockdown should increase susceptibility to DENv-2 infection. dsRNA-microinjected mosquitoes were exposed 24 h later to blood+DENv-2, a time in which the knockdown was approximately 80%. Because refractoriness in the Cali-MIB colony is not 100% fixed, it was expected that approximately 53% of the colony was susceptible and would allow DENv-2 to develop. Naïve mosquitoes or ds-Nautilus-injected mosquitoes maintained this proportion of susceptibility after exposure to DENv-2 (Fig. 4). In mosquitoes in which caspase-16 or Aedronc were knocked down, the proportion of mosquitoes that were susceptible to DENv-2 increased from the expected 53% to 64% and 80% respectively (Fig. 4) indicating that reducing the expression of pro-apoptotic genes increased pathogen success. Our attempts to silence AeIAP1, and convert the phenotype from susceptible to refractory resulted in spontaneous apoptosis and death, indicating that AeIAP1 is necessary to maintain cell viability in adult mosquitoes. These data support the growing body of literature that suggests that caspase activity in insects is regulated primarily after activation. Studies in both *Drosophila* and mosquito cell lines indicate that the primary apoptotic caspase in insects is Aedronc; that many cells experience chronic activation of Aedronc; and that insect cells, *in vitro* and *in vivo*, survive because they express IAP1 [78], [79], [80].

The development of resistance should depend on the pathological effects of DENv on mosquitoes and its effect on vector survival and fecundity [81], [82]. The effects of arboviruses on mosquitoes may be negative [83], [84], [85] or neutral [86], [87] and meta-analyses suggest that the effect of viruses on the vectors depends on the taxonomic groups studied and the mode of transmission [81]. Many studies have assessed immune responses of inbred mosquito colonies that have been laboratory acclimated for decades. The use of field-derived material such as Cali-MIB and Cali-S strains selected from the same feral populations with different responses to DENv-2 infection, provides us with the opportunity to identify natural mechanisms involved in refractoriness and will serve as a natural model to identify the functional role of defence related genes and immune pathways in the VC of these selected strains.

### 

#### Gene Accession numbers

The genes used in this study were Argonaute-2 (VectorBase: AAEL017251-RA), Aedronc (VectorBase AAEL011562-RA), Aedredd (VectorBase: AAEL014148-RA), Caspase-16, (VectorBase AAEL005956-RA), AeIAP1 (VectorBase: AAEL009074-RA), nautilus (Flybase: FBgn0002922) and *Ae. aegypti* ß-actin (AAEL001928-RA).

## Supporting Information

Table S1 **Sequence of Primers used in real time quantitative PCR reactions to determine gene expression in midguts of different strains of** ***Aedes aegypti*** **at different times after ingesting a bloodmeal containing Dengue-2 virus.**
(DOC)

## References

[1] Gubler DJ (2002) Epidemic dengue/dengue hemorrhagic fever as a public health, social and economic problem in the 21st century. Trends Microbiol 10: 100–103. 11827812 10.1016/s0966-842x(01)02288-0

[2] Gubler DJ (2004) The changing epidemiology of yellow fever and dengue, 1900 to 2003: full circle? Comp Immunol Microbiol Infect Dis 27: 319–330. 15225982 10.1016/j.cimid.2004.03.013

[3] Reiter P (2010) Yellow fever and dengue: a threat to Europe? Euro Surveill 15: 19509. 20403310

[4] Impoinvil DE, Ahmad S, Troyo A, Keating J, Githeko AK, et al (2007) Comparison of mosquito control programs in seven urban sites in Africa, the Middle East, and the Americas. Health Policy 83: 196–212. 17316882 10.1016/j.healthpol.2007.01.009 PMC2048658

[5] Mathur G, Sanchez-Vargas I, Alvarez D, Olson KE, Marinotti O, et al (2010) Transgene-mediated suppression of dengue viruses in the salivary glands of the yellow fever mosquito, Aedes aegypti. Insect Mol Biol 19: 753–763. 20738425 10.1111/j.1365-2583.2010.01032.x PMC2976824

[6] James AA (2007) Preventing the spread of malaria and dengue fever using genetically modified mosquitoes. J Vis Exp 231. 18979028 10.3791/231 PMC2557084

[7] Wise de Valdez MR, Nimmo D, Betz J, Gong HF, James AA, et al (2011) Genetic elimination of dengue vector mosquitoes. Proc Natl Acad Sci U S A 108: 4772–4775. 21383140 10.1073/pnas.1019295108 PMC3064365

[8] Frentiu FD, Robinson J, Young PR, McGraw EA, O'Neill SL (2010) Wolbachia-mediated resistance to dengue virus infection and death at the cellular level. PLoS One 5: e13398. 20976219 10.1371/journal.pone.0013398 PMC2955527

[9] Hoffmann AA, Montgomery BL, Popovici J, Iturbe-Ormaetxe I, Johnson PH, et al (2011) Successful establishment of Wolbachia in Aedes populations to suppress dengue transmission. Nature 476: 454–457. 21866160 10.1038/nature10356

[10] Walker T, Johnson PH, Moreira LA, Iturbe-Ormaetxe I, Frentiu FD, et al (2011) The wMel Wolbachia strain blocks dengue and invades caged Aedes aegypti populations. Nature 476: 450–453. 21866159 10.1038/nature10355

[11] Nurnberger T, Brunner F, Kemmerling B, Piater L (2004) Innate immunity in plants and animals: striking similarities and obvious differences. Immunol Rev 198: 249–266. 15199967 10.1111/j.0105-2896.2004.0119.x

[12] Medzhitov R, Janeway CA Jr (2002) Decoding the patterns of self and nonself by the innate immune system. Science 296: 298–300. 11951031 10.1126/science.1068883

[13] Hoffmann JA, Reichhart JM (2002) Drosophila innate immunity: an evolutionary perspective. Nat Immunol 3: 121–126. 11812988 10.1038/ni0202-121

[14] Hoffmann JA (2003) The immune response of Drosophila. Nature 426: 33–38. 14603309 10.1038/nature02021

[15] Lowenberger CA (2001) Form, function and phylogenetic relationships of mosquito immune peptides. Adv Exp Med Biol 484: 113–129. 11418977 10.1007/978-1-4615-1291-2_11

[16] Lowenberger CA, Kamal S, Chiles J, Paskewitz S, Bulet P, et al (1999) Mosquito-Plasmodium interactions in response to immune activation of the vector. Exp Parasitol 91: 59–69. 9920043 10.1006/expr.1999.4350

[17] Barón OL, Ursic Bedoya R, Lowenberger C, Ocampo C (2010) Differential gene Expression from midguts of refractory and susceptible lines of Aedes aegypti infected with Dengue-2 virus. J Insect Sci Vol 10: Article 41. 10.1673/031.010.4101 PMC3014741 20572793

[18] Behura SK, Gomez-Machorro C, Harker BW, deBruyn B, Lovin DD, et al (2011) Global cross-talk of genes of the mosquito Aedes aegypti in response to dengue virus infection. PLoS Negl Trop Dis 5: e1385. 22102922 10.1371/journal.pntd.0001385 PMC3216916

[19] Behura SK, Severson DW (2012) Intrinsic features of Aedes aegypti genes affect transcriptional responsiveness of mosquito genes to dengue virus infection. Infect Genet Evol 12: 1413–1418. 22579482 10.1016/j.meegid.2012.04.027 PMC3424315

[20] Bryant B, Blair CD, Olson KE, Clem RJ (2008) Annotation and expression profiling of apoptosis-related genes in the yellow fever mosquito, Aedes aegypti. Insect biochemistry and molecular biology 38: 331–345. 18252247 10.1016/j.ibmb.2007.11.012 PMC2258459

[21] Chauhan C, Behura SK, Debruyn B, Lovin DD, Harker BW, et al (2012) Comparative Expression Profiles of Midgut Genes in Dengue Virus Refractory and Susceptible Aedes aegypti across Critical Period for Virus Infection. PLoS One 7: e47350. 23077596 10.1371/journal.pone.0047350 PMC3471866

[22] Xi Z, Ramirez JL, Dimopoulos G (2008) The Aedes aegypti toll pathway controls dengue virus infection. PLoS pathogens 4: e1000098. 18604274 10.1371/journal.ppat.1000098 PMC2435278

[23] Cooper DM, Chamberlain CM, Lowenberger C (2009) Aedes FADD: A novel death domain-containing protein required for antibacterial immunity in the yellow fever mosquito, Aedes aegypti. Insect biochemistry and molecular biology 39: 47–54. 18977438 10.1016/j.ibmb.2008.09.011

[24] Sim S, Dimopoulos G (2010) Dengue virus inhibits immune responses in Aedes aegypti cells. PloS one 5: e10678. 20502529 10.1371/journal.pone.0010678 PMC2872661

[25] Souza-Neto JA, Sim S, Dimopoulos G (2009) An evolutionary conserved function of the JAK-STAT pathway in anti-dengue defense. Proceedings of the National Academy of Sciences of the United States of America 106: 17841–17846. 19805194 10.1073/pnas.0905006106 PMC2764916

[26] Bennett KE, Flick D, Fleming KH, Jochim R, Beaty BJ, et al (2005) Quantitative trait loci that control dengue-2 virus dissemination in the mosquito Aedes aegypti. Genetics 170: 185–194. 15781707 10.1534/genetics.104.035634 PMC1449711

[27] Bennett KE, Olson KE, Munoz MD, Fernandez-Salas I, Farfan-Ale JA, et al (2002) Variation in vector competence for dengue 2 virus among 24 collections of Aedes aegypti from Mexico and the United States. American Journal of Tropical Medicine and Hygiene 67: 85–92. 12363070 10.4269/ajtmh.2002.67.85

[28] Black WCt, Bennett KE, Gorrochotegui-Escalante N, Barillas-Mury CV, Fernandez-Salas I, et al (2002) Flavivirus susceptibility in Aedes aegypti. Archives of medical research 33: 379–388. 12234528 10.1016/s0188-4409(02)00373-9

[29] Bosio CF, Fulton RE, Salasek ML, Beaty BJ, Black WCt (2000) Quantitative trait loci that control vector competence for dengue-2 virus in the mosquito Aedes aegypti. Genetics 156: 687–698. 11014816 10.1093/genetics/156.2.687 PMC1461298

[30] Colpitts TM, Cox J, Vanlandingham DL, Feitosa FM, Cheng G, et al (2011) Alterations in the Aedes aegypti Transcriptome during Infection with West Nile, Dengue and Yellow Fever Viruses. PLoS Pathog 7: e1002189. 21909258 10.1371/journal.ppat.1002189 PMC3164632

[31] Lambrechts L (2011) Quantitative genetics of Aedes aegypti vector competence for dengue viruses: towards a new paradigm? Trends Parasitol 10.1016/j.pt.2010.12.001 21215699

[32] Schneider JR, Chadee DD, Mori A, Romero-Severson J, Severson DW (2011) Heritability and adaptive phenotypic plasticity of adult body size in the mosquito Aedes aegypti with implications for dengue vector competence. Infect Genet Evol 11: 11–16. 21070891 10.1016/j.meegid.2010.10.019 PMC3005082

[33] Ocampo CB, Wesson DM (2004) Population dynamics of Aedes aegypti from a dengue hyperendemic urban setting in Colombia. Am J Trop Med Hyg 71: 506–513. 15516650

[34] Caicedo PA, Barón OL, Pérez M, Alexander N, Lowenberger C, et al (2012) Selection of Aedes aegypti strains susceptible or refractory to Dengue-2 virus. The Canadian Entomologist In Press.

[35] Hengartner MO (2000) The biochemistry of apoptosis. Nature 407: 770–776. 11048727 10.1038/35037710

[36] Thornberry NA (1998) Caspases: key mediators of apoptosis. Chem Biol 5: R97–103. 9578633 10.1016/s1074-5521(98)90615-9

[37] Benedict CA, Norris PS, Ware CF (2002) To kill or be killed: viral evasion of apoptosis. Nat Immunol 3: 1013–1018. 12407409 10.1038/ni1102-1013

[38] Raff M (1998) Cell suicide for beginners. Nature 396: 119–122. 9823889 10.1038/24055

[39] Clarke TE, Clem RJ (2003) Insect defenses against virus infection: the role of apoptosis. Int Rev Immunol 22: 401–424. 12959752 10.1080/08830180305215

[40] Zhou L, Jiang G, Chan G, Santos CP, Severson DW, et al (2005) Michelob_x is the missing inhibitor of apoptosis protein antagonist in mosquito genomes. EMBO reports 6: 769–774. 16041319 10.1038/sj.embor.7400473 PMC1369144

[41] Bowers DF, Coleman CG, Brown DT (2003) Sindbis virus-associated pathology in Aedes albopictus (Diptera: Culicidae). J Med Entomol 40: 698–705. 14596286 10.1603/0022-2585-40.5.698

[42] Girard YA, Popov V, Wen J, Han V, Higgs S (2005) Ultrastructural study of West Nile virus pathogenesis in Culex pipiens quinquefasciatus (Diptera: Culicidae). J Med Entomol 42: 429–444. 15962797 10.1093/jmedent/42.3.429

[43] Vaidyanathan R, Scott TW (2006) Apoptosis in mosquito midgut epithelia associated with West Nile virus infection. Apoptosis 11: 1643–1651. 16820968 10.1007/s10495-006-8783-y

[44] Cooper DM, Pio F, Thi EP, Theilmann D, Lowenberger C (2007) Characterization of Aedes Dredd: a novel initiator caspase from the yellow fever mosquito, Aedes aegypti. Insect Biochem Mol Biol 37: 559–569. 17517333 10.1016/j.ibmb.2007.03.005

[45] Cooper DM, Thi EP, Chamberlain CM, Pio F, Lowenberger C (2007) Aedes Dronc: a novel ecdysone-inducible caspase in the yellow fever mosquito, Aedes aegypti. Insect Mol Biol 16: 563–572. 17725799 10.1111/j.1365-2583.2007.00758.x

[46] Higgs S, Traul D, Davis BS, Kamrud KI, Wilcox CL, et al (1996) Green fluorescent protein expressed in living mosquitoes-without the requirement of transformation. Biotechniques 21: 660–664. 8891217 10.2144/96214st03

[47] Higgs S, Olson KE, Kamrud KI, Powers A, Beaty B (1997) Viral expression systems and viral infections in insects. . in The Molecular Biology of Insect Disease Vectors- A method manual Ed By JM Crampton, CB Beard and C Louis Chapman & Hall, Printed in Great Britain by the University Press, Cambridge 578p

[48] Wallis GP, Aitken THG, Beaty BJ, Lorenz L, Amato GD, et al (1985) Selection for Susceptibility and Refractoriness of Aedes-Aegypti to Oral Infection with Yellow-Fever Virus. American Journal of Tropical Medicine and Hygiene 34: 1225–1231. 3834805 10.4269/ajtmh.1985.34.1225

[49] Lanciotti RS, Calisher CH, Gubler DJ, Chang GJ, Vorndam AV (1992) Rapid detection and typing of dengue viruses from clinical samples by using reverse transcriptase-polymerase chain reaction. J Clin Microbiol 30: 545–551. 1372617 10.1128/jcm.30.3.545-551.1992 PMC265106

[50] Cooper DM (2008) Apoptosis and immunity: characterizing the cell death machinery in the Yellow Fever mosquito, Aedes aegypti.:. Simon Fraser University

[51] Campbell CL, Keene KM, Brackney DE, Olson KE, Blair CD, et al (2008) Aedes aegypti uses RNA interference in defense against Sindbis virus infection. BMC microbiology 8: 47. 18366655 10.1186/1471-2180-8-47 PMC2278134

[52] Cirimotich CM, Scott JC, Phillips AT, Geiss BJ, Olson KE (2009) Suppression of RNA interference increases alphavirus replication and virus-associated mortality in Aedes aegypti mosquitoes. BMC microbiology 9: 49. 19265532 10.1186/1471-2180-9-49 PMC2660349

[53] Sanchez-Vargas I, Scott JC, Poole-Smith BK, Franz AW, Barbosa-Solomieu V, et al (2009) Dengue virus type 2 infections of Aedes aegypti are modulated by the mosquito's RNA interference pathway. PLoS pathogens 5: e1000299. 19214215 10.1371/journal.ppat.1000299 PMC2633610

[54] Livak KJ, Schmittgen TD (2001) Analysis of relative gene expression data using real-time quantitative PCR and the 2(−Delta Delta C(T)) Method. Methods (San Diego, Calif 25: 402–408. 10.1006/meth.2001.1262 11846609

[55] Schmittgen TD, Livak KJ (2008) Analyzing real-time PCR data by the comparative C(T) method. Nature protocols 3: 1101–1108. 18546601 10.1038/nprot.2008.73

[56] Tardieux I, Poupel O, Lapchin L, Rodhain F (1991) Analysis of Inheritance of Oral-Susceptibility of Aedes-Aegypti (Diptera, Culicidae) to Dengue-2 Virus Using Isofemale Lines. Journal of Medical Entomology 28: 518–521. 1941912 10.1093/jmedent/28.4.518

[57] Ferrandon D, Imler JL, Hetru C, Hoffmann JA (2007) The Drosophila systemic immune response: sensing and signalling during bacterial and fungal infections. Nat Rev Immunol 7: 862–874. 17948019 10.1038/nri2194

[58] Lemaitre B, Hoffmann J (2007) The host defense of Drosophila melanogaster. Annu Rev Immunol 25: 697–743. 17201680 10.1146/annurev.immunol.25.022106.141615

[59] Dostert C, Jouanguy E, Irving P, Troxler L, Galiana-Arnoux D, et al (2005) The Jak-STAT signaling pathway is required but not sufficient for the antiviral response of drosophila. Nat Immunol 6: 946–953. 16086017 10.1038/ni1237

[60] Sanders HR, Foy BD, Evans AM, Ross LS, Beaty BJ, et al (2005) Sindbis virus induces transport processes and alters expression of innate immunity pathway genes in the midgut of the disease vector, Aedes aegypti. Insect Biochem Mol Biol 35: 1293–1307. 16203210 10.1016/j.ibmb.2005.07.006

[61] Teixeira L, Ferreira A, Ashburner M (2008) The bacterial symbiont Wolbachia induces resistance to RNA viral infections in Drosophila melanogaster. PLoS Biol 6: e2. 10.1371/journal.pbio.1000002 PMC2605931 19222304

[62] Ramirez JL, Dimopoulos G (2010) The Toll immune signaling pathway control conserved anti-dengue defenses across diverse Ae. aegypti strains and against multiple dengue virus serotypes. Developmental and comparative immunology 34: 625–629. 20079370 10.1016/j.dci.2010.01.006 PMC2917001

[63] Sessions OM, Barrows NJ, Souza-Neto JA, Robinson TJ, Hershey CL, et al (2009) Discovery of insect and human dengue virus host factors. Nature 458: 1047–1050. 19396146 10.1038/nature07967 PMC3462662

[64] Paquette N, Broemer M, Aggarwal K, Chen L, Husson M, et al (2010) Caspase-mediated cleavage, IAP binding, and ubiquitination: linking three mechanisms crucial for Drosophila NF-kappaB signaling. Mol Cell 37: 172–182. 20122400 10.1016/j.molcel.2009.12.036 PMC2819219

[65] Georgel P, Naitza S, Kappler C, Ferrandon D, Zachary D, et al (2001) Drosophila immune deficiency (IMD) is a death domain protein that activates antibacterial defense and can promote apoptosis. Dev Cell 1: 503–514. 11703941 10.1016/s1534-5807(01)00059-4

[66] Luplertlop N, Surasombatpattana P, Patramool S, Dumas E, Wasinpiyamongkol L, et al (2011) Induction of a peptide with activity against a broad spectrum of pathogens in the Aedes aegypti salivary gland, following Infection with Dengue Virus. PLoS Pathog 7: e1001252. 21249175 10.1371/journal.ppat.1001252 PMC3020927

[67] Fragkoudis R, Attarzadeh-Yazdi G, Nash AA, Fazakerley JK, Kohl A (2009) Advances in dissecting mosquito innate immune responses to arbovirus infection. J Gen Virol 90: 2061–2072. 19570957 10.1099/vir.0.013201-0

[68] Tardieux I, Poupel O, Lapchin L, Rodhain F (1990) Variation among Strains of Aedes-Aegypti in Susceptibility to Oral Infection with Dengue Virus Type-2. American Journal of Tropical Medicine and Hygiene 43: 308–313. 2221225 10.4269/ajtmh.1990.43.308

[69] Liu B, Becnel JJ, Zhang Y, Zhou L (2011) Induction of reaper ortholog mx in mosquito midgut cells following baculovirus infection. Cell Death Differ 18: 1337–1345. 21331076 10.1038/cdd.2011.8 PMC3172108

[70] Li Q, Li H, Blitvich BJ, Zhang J (2007) The Aedes albopictus inhibitor of apoptosis 1 gene protects vertebrate cells from bluetongue virus-induced apoptosis. Insect Mol Biol 16: 93–105. 17257212 10.1111/j.1365-2583.2007.00705.x

[71] Girard YA, Schneider BS, McGee CE, Wen J, Han VC, et al (2007) Salivary gland morphology and virus transmission during long-term cytopathologic West Nile virus infection in Culex mosquitoes. Am J Trop Med Hyg 76: 118–128. 17255239

[72] Salazar MI, Richardson JH, Sanchez-Vargas I, Olson KE, Beaty BJ (2007) Dengue virus type 2: replication and tropisms in orally infected Aedes aegypti mosquitoes. BMC Microbiol 7: 9. 17263893 10.1186/1471-2180-7-9 PMC1797809

[73] Wang H, Blair CD, Olson KE, Clem RJ (2008) Effects of inducing or inhibiting apoptosis on Sindbis virus replication in mosquito cells. The Journal of general virology 89: 2651–2661. 18931060 10.1099/vir.0.2008/005314-0 PMC2603079

[74] Wang H, Clem RJ (2011) The role of IAP antagonist proteins in the core apoptosis pathway of the mosquito disease vector Aedes aegypti. Apoptosis 16: 235–248. 21274634 10.1007/s10495-011-0575-3 PMC3197237

[75] Franz AW, Sanchez-Vargas I, Adelman ZN, Blair CD, Beaty BJ, et al (2006) Engineering RNA interference-based resistance to dengue virus type 2 in genetically modified Aedes aegypti. Proceedings of the National Academy of Sciences of the United States of America 103: 4198–4203. 16537508 10.1073/pnas.0600479103 PMC1449670

[76] Hess AM, Prasad AN, Ptitsyn A, Ebel GD, Olson KE, et al (2011) Small RNA profiling of Dengue virus-mosquito interactions implicates the PIWI RNA pathway in anti-viral defense. BMC Microbiol 11: 45. 21356105 10.1186/1471-2180-11-45 PMC3060848

[77] Cirimotich CM, Dong Y, Garver LS, Sim S, Dimopoulos G (2010) Mosquito immune defenses against Plasmodium infection. Dev Comp Immunol 34: 387–395. 20026176 10.1016/j.dci.2009.12.005 PMC3462653

[78] Igaki T, Yamamoto-Goto Y, Tokushige N, Kanda H, Miura M (2002) Down-regulation of DIAP1 triggers a novel Drosophila cell death pathway mediated by Dark and DRONC. J Biol Chem 277: 23103–23106. 12011068 10.1074/jbc.C200222200

[79] Muro I, Hay BA, Clem RJ (2002) The Drosophila DIAP1 protein is required to prevent accumulation of a continuously generated, processed form of the apical caspase DRONC. J Biol Chem 277: 49644–49650. 12397080 10.1074/jbc.M203464200

[80] Liu Q, Clem RJ (2011) Defining the core apoptosis pathway in the mosquito disease vector Aedes aegypti: the roles of iap1, ark, dronc, and effector caspases. Apoptosis 16: 105–113. 21107703 10.1007/s10495-010-0558-9 PMC6029261

[81] Lambrechts L, Scott TW (2009) Mode of transmission and the evolution of arbovirus virulence in mosquito vectors. Proc Biol Sci 276: 1369–1378. 19141420 10.1098/rspb.2008.1709 PMC2660968

[82] Lambrechts L (2010) Dissecting the genetic architecture of host-pathogen specificity. PLoS Pathog 6. 10.1371/journal.ppat.1001019 PMC2916863 20700450

[83] Mahmood F, Reisen WK, Chiles RE, Fang Y (2004) Western equine encephalomyelitis virus infection affects the life table characteristics of Culex tarsalis (Diptera: Culicidae). J Med Entomol 41: 982–986. 15535632 10.1603/0022-2585-41.5.982

[84] Moncayo AC, Edman JD, Turell MJ (2000) Effect of eastern equine encephalomyelitis virus on the survival of Aedes albopictus, Anopheles quadrimaculatus, and Coquillettidia perturbans (Diptera: Culicidae). J Med Entomol 37: 701–706. 11004781 10.1603/0022-2585-37.5.701

[85] Moncayo AC, Fernandez Z, Ortiz D, Diallo M, Sall A, et al (2004) Dengue emergence and adaptation to peridomestic mosquitoes. Emerg Infect Dis 10: 1790–1796. 15504265 10.3201/eid1010.030846 PMC3323252

[86] Putnam JL, Scott TW (1995) Blood-feeding behavior of dengue-2 virus-infected Aedes aegypti. Am J Trop Med Hyg 52: 225–227. 7694963 10.4269/ajtmh.1995.52.225

[87] Berry WJ, Rowley WA, Clarke JL 3rd, Swack NS, Hausler WJ Jr (1987) Spontaneous flight activity of Aedes trivittatus (Diptera: Culicidae) infected with trivittatus virus (Bunyaviridae: California serogroup). J Med Entomol 24: 286–289. 3585922 10.1093/jmedent/24.3.286

