# Supplementary material for: Differential Expression of Apoptosis Related Genes in Selected Strains of Aedes aegypti with Different Susceptibilities to Dengue Virus
Source: PLoS One. 2013 Apr 10;8(4):e61187. doi: 10.1371/journal.pone.0061187 (PMC3622604; doi:10.1371/journal.pone.0061187)
Supplement: Table S1 — Sequence of Primers used in real time quantitative PCR reactions to determine gene expression in midguts of different strains of Aedes aegypti at different times after ingesting a bloodmeal containing Dengue-2 virus. (DOC) [file pone.0061187.s001.doc]

| Primer name | Abbreviation | Sequence (5’ to 3’) |
| --- | --- | --- |
| ß-actin forward | qActF | AAGGCTAACCGTGAGAAGATGAC |
| ß-actin Reverse | qActR | GATTGGGACAGTGTGGGAGAC |
| Caspase 16 Forward | CaspF | TCCGCTATCTTCATATTGTATCCTTTG |
| Caspase 16 Reverse | CaspR | GACCCGCCACTGTATCTCTG |
| Argonaute 2 Forward | ArgoF | CCGTTCTGGACATGACTTGC |
| Argonaute 2 Reverse | ArgoR | CACAGCTCATGGTTGCTTCC |
| AeDronc Forward | DroncF | CAACTTTCCAACTGCCTATAAATTGC |
| AeDronc Reverse | DroncR | CTCCACCGTATCGTTATTGTTCTTAG |
| AeIAP1 Forward | IAP1F | CTGAAACTAATGAAGGGCGAAGC |
| AeIAP1 Reverse | IAP1R | TTGAGATGACTGAAGCGAGGATG |
| AeDredd Forward | DreddF | GAACATTGAAAGCAACTTCAACCG |
| AeDredd Reverse | DreddR | GCCATTTTGTCCACCTCGG |

**Table S1**. Sequence of Primers used in real time quantitative PCR reactions to determine gene expression in midguts of different strains of *Aedes aegypti* at different times after ingesting a bloodmeal containing Dengue-2 virus.
